# Ultrasound-guided needle release of the transverse carpal ligament with and without corticosteroid injection for the treatment of carpal tunnel syndrome

**DOI:** 10.1186/s13018-018-0771-8

**Published:** 2018-04-03

**Authors:** Xuan-Yan Guo, Mao-Xiang Xiong, Man Lu, Xue-Qing Cheng, Yan-Yan Wu, Shi-Yin Chen, Kai Chen, Qiao-Dan Zhou, Lei Wang, Li Tan, Jie-Rong Quan, Fan-Ding He, Qin Chen

**Affiliations:** 1 Ultrasonic Department, Sichuan Academy of Medical Sciences & Sichuan Provincial People’s Hospital, School of Medicine, University of Electronic Science and Technology of China, No. 32, West Section 2, Yihuan Road, Qingyang District, Chengdu, 610072 People’s Republic of China; 2 Psychiatry Department, Chengdu Mental Health Center, The Fourth People’s Hospital of Chengdu, Chengdu, 610036 People’s Republic of China; 3 Ultrasonic Department, Sichuan Cancer Hospital Institute, Sichuan Cancer Center, School of Medicine, University of Electronic Science and Technology of China, Chengdu, 610041 People’s Republic of China; 4 Department of Chinese medicine orthopaedics, Sichuan Academy of Medical Sciences & Sichuan Provincal People’s Hospital, School of Medicine, University of Electronic Science and Technology of China, Chengdu, 610072 People’s Republic of China

**Keywords:** Carpal tunnel syndrome, Ultrasound, Corticosteroid injection

## Abstract

**Background:**

To compare the clinical effectiveness of ultrasound-guided needle release of the transverse carpal ligament (TCL) with and without corticosteroid injection in carpal tunnel syndrome (CTS).

**Methods:**

From June 2016 to June 2017, 49 CTS patients (50 wrists) were included in this study. Twenty-five wrists were treated with ultrasound-guided needle release of the TCL plus corticosteroid injection (group A), and 25 wrists were treated with single ultrasound-guided needle release of the TCL (group B). The following parameters were assessed and compared including postprocedure results according to relief of symptoms, ultrasound parameters (cross-sectional area of the median nerve at the levels of pisiform, flattening ratio of median nerve at the levels of the hamate bone, and the thicknesses of TCL on the cross-section at the level of the hamate bone), and electrophysiological parameters (distal motor latency and sensory conduction velocity).

**Results:**

Group A had higher overall excellent and good rate 3 months after the procedure than group B (84 vs 52%, *P* < 0.05). There were significant differences regarding the above ultrasonic and electrophysiological parameters between the baseline and postprocedure values in both groups (all *P* < 0.05). There were significant differences regarding the postprocedure values of above ultrasonic and electrophysiological parameters between the two groups (all *P* < 0.05). No complications such as infection or tendon rupture were noted. No procedures were converted to the open release.

**Conclusions:**

Both techniques are effective in treating CTS. Ultrasound-guided needle release of the TCL with corticosteroid injection had better treatment benefits than single ultrasound-guided needle release of the TCL in treating CTS.

## Background

Carpal tunnel syndrome (CTS) is the most common form of peripheral compressive neuropathy [1]. It is caused by compression of the median nerve at the wrist [2]. The common symptoms include pain, paresthesia, numbness, sleep disturbance, and weakness of the hand [3]. CTS is more prevalent in females than in males [4]. Its diagnosis mainly depended on the history and physical examination and can be confirmed by electrophysiological testing [5]. Various strategies are available for the management of CTS. These strategies can be categorized into two types: conservative and surgical. The choice of the treatment depends on the severity of the symptoms, chronicity of the disease, and the patient’s preference [6]. Conservative treatments are usually recommended as an initial treatment for patients at early and middle stages [7, 8], while the surgical treatments are indicated for patients at advanced state. The conservative treatment for CTS include activity modification, wrist splinting, oral medications and vitamins, exercise, local corticosteroid injections into the carpal canal, or other managements (such as laser therapy, therapeutic ultrasound, or acupuncture) [4, 9, 10].

Local corticosteroid injections have been widely used for the short-term treatment of CTS. A systematic review of 12 studies with 671 participants demonstrated that corticosteroid injections give better clinical improvement then placebo injection for 1 month after injection, and greater improvement than oral corticosteroids for 3 months after injection [11]. The development of high-resolution ultrasound scanning allows detailed visualization of the anatomical structures of carpal tunnel, including the median nerve, flexor tendons, and transverse carpal ligament (flexor retinaculum) as well as at-risk structures, such as the ulnar artery and the superficial palmar arch [12].

Conservative treatments are mainly indicated for early-to-middle-stage CTS. If conservative treatments failed to alleviate symptoms sufficiently, patients will have to select surgical treatments previously. However, surgical treatments are associated with relatively larger trauma, higher costs, and longer time to restore to normal. We attempt to seek a minimally invasive, convenient, and cost-saving method for this kind of patients. Real-time sonography has opened up new possibilities for percutaneous treatments beyond traditional corticosteroid injections. Thus, in this study, we introduced a minimally invasive method of ultrasound-guided needle release of the TCL with corticosteroid injection. However, for certain patients such as those with severe hypertension, diabetes mellitus, early pregnancy, or those sensitive to corticosteroids, the corticosteroid injection are contraindicated. Thus, we hypothesized that single ultrasound-guided release of the TCL would be safe and effective for CTS in this selected patients. The purpose of this study was to compare the clinical effectiveness of ultrasound-guided needle release of the TCL with and without corticosteroid injection in early-to-middle-stage CTS.

## Methods

This study was approved by the ethic committee of Sichuan Academy of Medical Sciences & Sichuan Provincial People’s Hospital and conducted in accordance with the Helsinki Declaration. Written informed consent was obtained from all subjects. From June 2016 to June 2017, 49 early-to-middle-stage CTS patients (50 wrists) treated by ultrasound-guided needle release of the TCL with and without corticosteroid injection at our hospital were included in this study. The clinical diagnosis of CTS was made based on a clinical diagnosis including history and physical examination (such as pain, numbness, paresthesia, muscle force, and night waking), ultrasonic evaluation, and electrophysiological confirmation.

Exclusion criteria were CTS at advanced stage, polyneuritis, poor general physical condition, carpal deformity, carpal fractures, wrist foreign body such as tumors and cyst, and previous history of wrist surgery.

### Instruments

Ultrasonic examinations were performed by two senior radiologists with more than 10 years’ experience in ultrasound by using the Philips iU22 scanner with a 5- to 12-MHz linear array transducer (Philips Healthcare Solutions, Bothell, WA, USA) and Philips Elite scanner (Philips Healthcare Solutions,). Standard 22-gauge needles (Becton Dickinson SA, Spain) were used for the local anesthesia and preparation of the puncture wound.

### Procedures

**Fig. 1:**
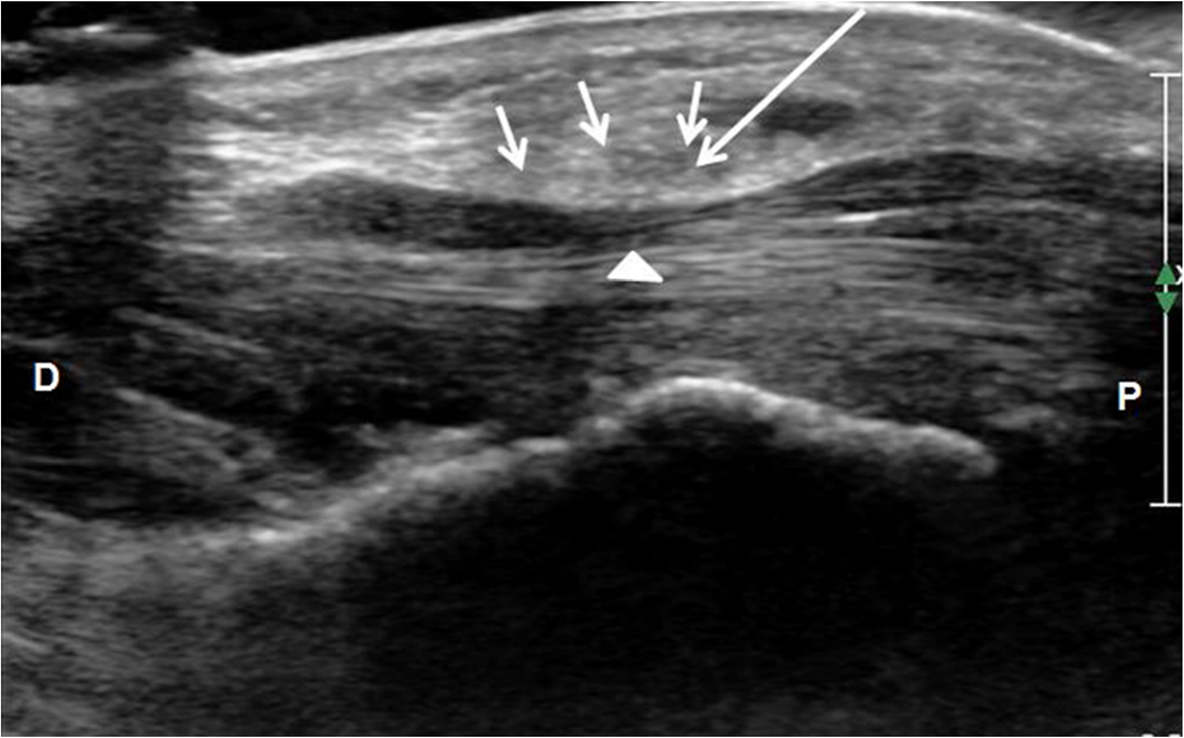
Longitudinal ultrasonic image of carpal tunnel. Long arrow suggests the entry needle route of release. Triangle arrow suggests median nerve compression. Short arrow suggests thickened TCL. *P* proximal end; *D* distal end

In the experiment group, all patients were treated by using ultrasound-guided percutaneous needle release of the TCL with corticosteroid injection. The patient was placed in the sitting position with their forearm and fingers resting on a table. The affected wrist was then positioned to the side with the palm facing upwards. A pillow was put below the affected wrist. The transducer was coated with Standard acoustic coupling agent (Ambition TC, Chongqing, People’s Republic of China) and then enclosed in a sterilized covering and a surgical glove. The transducer was then placed in contact with the affected wrist. Transverse scans through the carpal tunnel was performed to visualize the key anatomic structures such as TCL, median nerve, deep or superficial digital flexor tendons, flexor pollicis longus tendon, ulnar artery, and the bone landmarks, i.e., the scaphoid, pisiform bone. Then, the cross-sectional area of the median nerve at the level of pisiform, the left-right diameter and anteroposterior diameter at the level of the hamate bone, and the thicknesses of TCL on the cross-section at the level of the hamate bone were measured. The median nerve was then longitudinally scanned and the superficial TCL was localized. On the longitudinal image, the needle entry point was marked at about 1–2 cm proximal to the hamate bone plane (Fig. 1). After wiping the skin with ethyl alcohol, a 22-gauge hypodermic needle was advanced at a 15–20° slope angle to imaging plane. Under the guidance of continuous ultrasound, 4 mL of local anesthetics including 2% lidocaine (2 ml) and 0.9% sodium chloride injection (2 ml) was injected. After the local anesthesia, the needle was utilized to repeatedly perforate the TCL in a direction parallel to the median nerve under the guidance of continuous ultrasound. During the process of needle puncture, care should be taken to avoid the injury to the median nerve. The ligament will be suggested to be adequately released if the needle was able to passed easily through the ligament. Then, the syringe was changed. 0.5 ml of compound betamethasone (5 mg betamethasone dipropionate and 2 mg betamethasone disodium phosphate per milliliter) (Schering-Plough Labo NV Belgium) was injected into the TCL. Finally, the needle was withdrawn, and the needle entry point was pressed for 3–5 min.

In the control group, the same procedures were performed until the completeness of the puncture of TCL. In this group, corticosteroid drugs were not injected.

### Postprocedure management

After the procedure, the puncture wound of the inlet was covered with a band-aid for 24 h and the affected wrist was immobilized for 3 days. Then, non-steroidal anti-inflammatory drugs (NSAIDs) were taken, and physical, such as ice compresses, and thermal therapies were started.

### Outcome measurements

Patients were invited to return to our hospital 3 months after the procedures. The patients were asked to grade the outcome of the procedure according to relief of symptoms referencing to a previous report by Kelly et al. [13]: (1) excellent, complete relief of symptoms and recovery of function, (2) good, obvious relief of symptoms and existence of occasional minor symptoms, (3) fair, some constant or annoying symptoms, and (4) poor, symptoms unchanged or worse. The assessments were performed by two experienced senior operators.

#### Ultrasonic evaluation

Ultrasonic evaluations were conducted by the two senior operators with more than 10 years of experience in sonography, who was blinded as to which hand had received treatment for CTS. Ultrasonic measurements of the median nerve were performed while the patient is seated with the dorsal aspect of the hand and affected wrist lying on an examining table with a pillow beneath to the wrist.

**Fig. 2:**
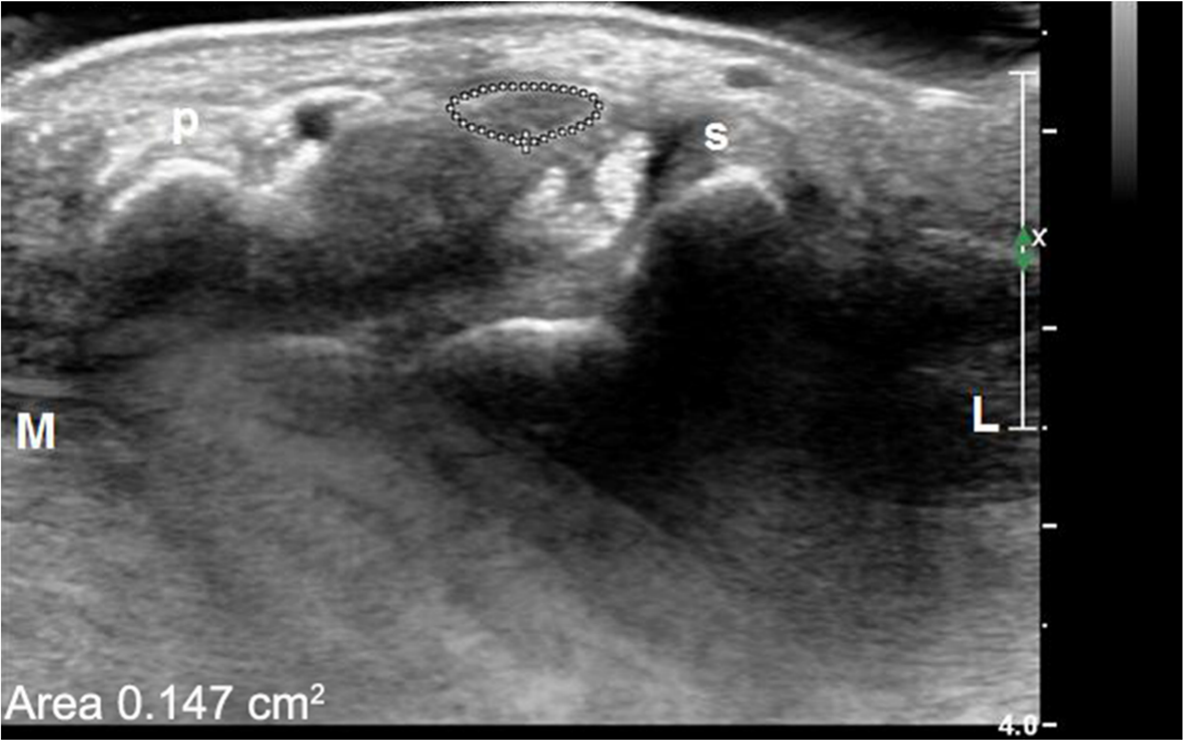
Transverse ultrasonic image of the proximal carpal tunnel at the level of the pisiform bone showed thickened median nerve and reduced echo. The area of the median nerve was 0.147 cm^2^. *S* scaphoid bone; *P* pisiform bone; *L* lateral; *M* medial

**Fig. 3:**
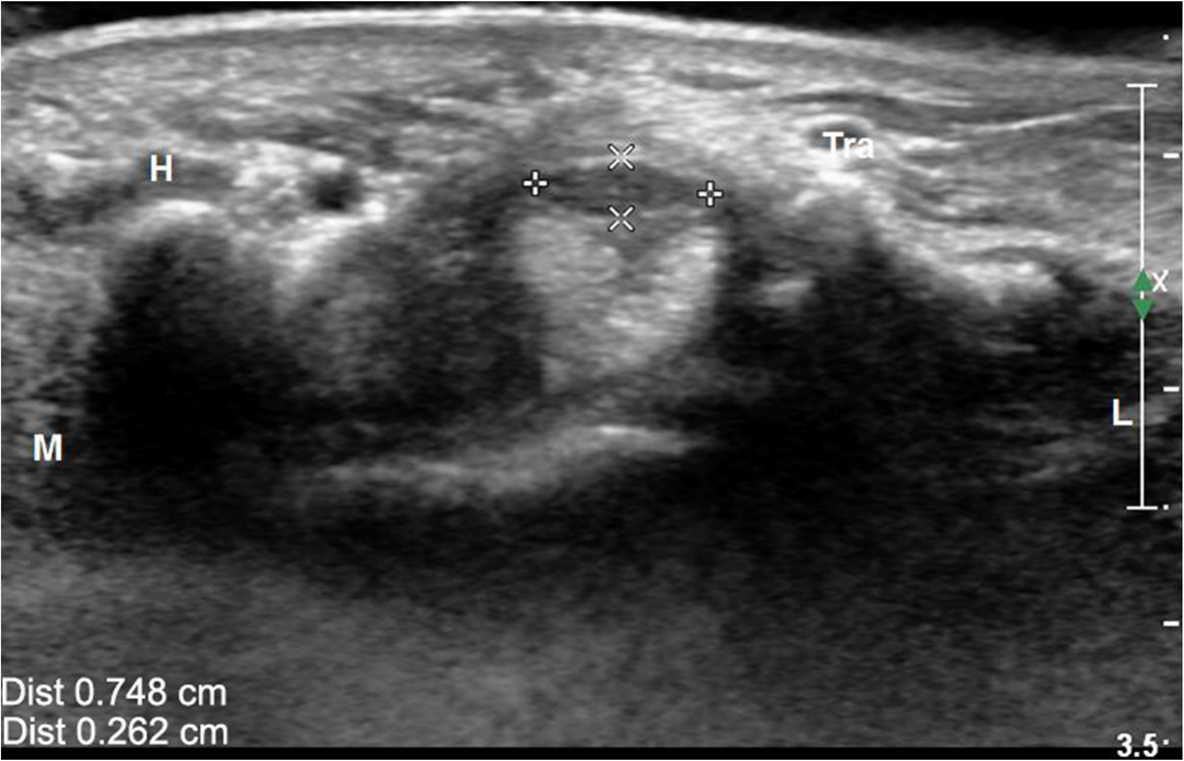
Transverse ultrasonic image of the distal carpal tunnel at the level of the pisiform bone showed median nerve compression. The gap between “++” suggests the left-right diameter of the median nerve. The gap between “xx” suggests the anteroposterior diameter of the median nerve. The left-right diameter and anteroposterior diameter of the median nerve at the level of the hamate bone was 0.748 and 0.262 cm, respectively. *H* hamate bone; *Tra* trapezium bone; *L* lateral; *M* medial

**Fig. 4:**
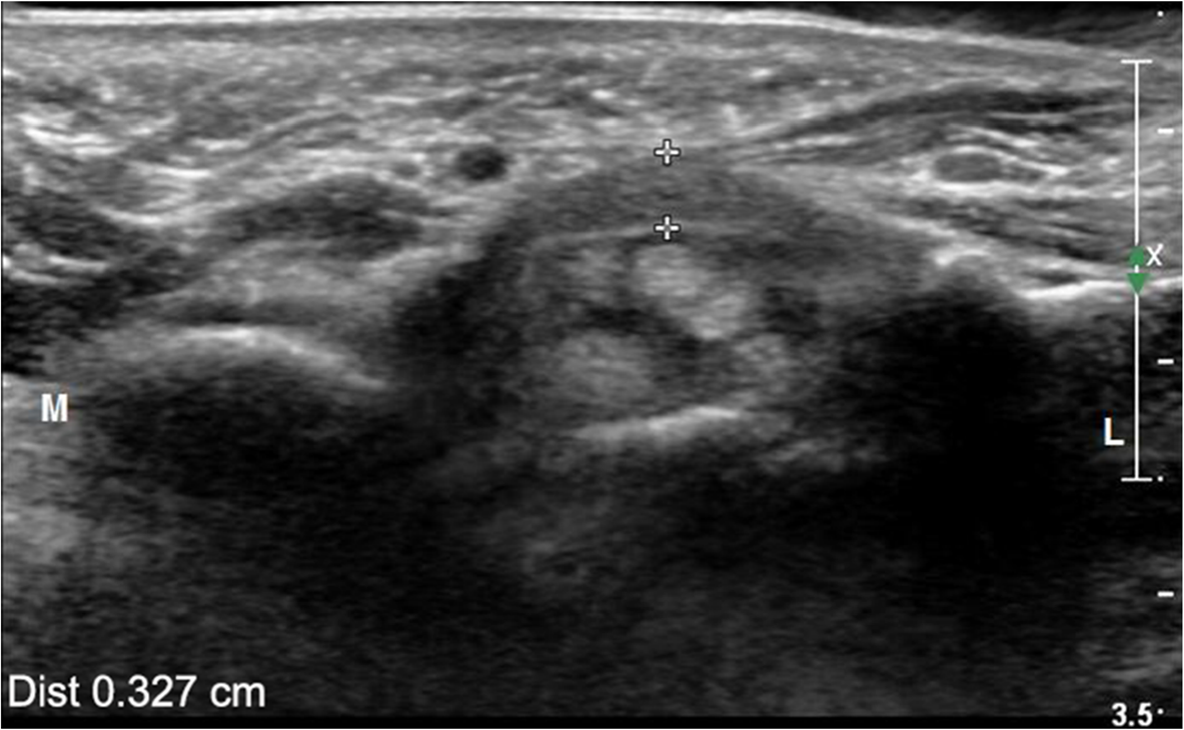
Transverse ultrasonic image of the distal carpal tunnel at the level of the hamate bone showed median nerve compression. The gap between “++” suggests thicknesses of TCL (0.327 cm) on the cross-section at the level of the hamate bone. *L* lateral; *M* medial

Axial views of the median nerve were obtained using a 7.5-MHz linear array transducer. The cross-sectional area of the median nerve at the level of pisiform, the left-right diameter and anteroposterior diameter at the level of the hamate bone, and the thicknesses of TCL on the cross-section at the level of the hamate bone were measured at baseline and 3 months after the procedure. The cross-sectional area of the median nerve at the level of pisiform was measured directly with area measurement software, using a continuous boundary trace (Fig. 2). Each measurement was repeated three times by one researcher, and the mean value was quantified. The left-right diameter and anteroposterior diameter at the level of the hamate bone is shown in Fig. 3. Then, we calculated the flattening ratio of median nerve by the ratio of left-right diameter/anteroposterior diameter at the level of the hamate bone. The thicknesses of TCL on the cross-section at the level of the hamate bone was calculated directly using electronic on-screen calipers just proximal to the tunnel where the nerve was thickest (proximal diameter) and within the carpal tunnel where the nerve was most flattened (distal diameter) (Fig. 4).

Finally, any potential complications such as infection or nerve damage were all recorded.

#### Electrophysiological evaluations

Distal motor latency (DML) and sensory conduction velocity (SCV) were measured at baseline and 3 month after the procedure. The assessments were performed by the two experienced senior operators.

### Statistical analysis

Data analyses were performed by using SPSS software, version 19.0 (SPSS Inc, Chicago, IL). Quantitative data were expressed as means ± standard deviations (SD) and were compared using student t test. Qualitative data were expressed as number and percentage. Ordered data was compared by using the Mann-Whitney rank sum test. A *P* value of less than 0.05 was considered as statistically different.

## Results

A total of 49 patients (50 wrists) with CTS were included in this study. Among these patents, there were 12 males and 38 females. The mean age was 49.58 ± 7.56 years. The mean duration of disease was 20.12 ± 8.35 months (range, 7-36 months). Among these patients, 25 wrists treated with ultrasound-guided needle release of the TCL plus corticosteroid injection (group A) and 25 wrists treated with single ultrasound-guided needle release of the TCL (group B). The detailed clinical and baseline characteristics were given in Table 1.

**Table 1:** Baseline characteristics in two groups

	Group A (*n* = 25 wrists)	Group B (*n* = 25 wrists)	*P* values
Cases	24 cases	25 cases	NS
Age, years	50.52 ± 8.30	48.64 ± 6.78	0.385
Sex			
Male	5	8	0.376
Female	19	17	
BMI, kg/m^2^	22.48 ± 2.10	22.04 ± 2.37	0.493
Mean duration of disease, months	20.92.16 ± 9.18	19.32 ± 7.54	0.504
Lesion site			
Right	17	20	0.508
Left	6	5	
Bilateral	1	0	

Group A, ultrasound-guided needle release of the transverse carpal ligament plus corticosteroid injection; Group B, single ultrasound-guided needle release of the transverse carpal ligament; BMI, Body Mass Index

### Postprocedure results

Postprocedure results were excellent in 14 wrists (57.1%), good in 7 wrists (25%), fair in 3 wrists (14.3%), and poor in 1 wrist (3.6%) in group A, and excellent in 7 wrists (25%), good in 6 wrists (21.4%), fair in 9 wrists (39.3%), and poor in 3 wrists (14.3%) in group B, respectively. The overall excellent and good rate was 84.0% in group A and 52.0% in group B (*P* < 0.05). In addition, no complications such as infection or tendon rupture were noted. No procedures were converted to the open release.

### Ultrasonic results

Ultrasonic results at baseline and 3 months after the procedure in two groups were shown in Table 2. Prior to the procedure, there were no statistically significant differences with regard to the baseline cross-sectional area of the MN at the level of the pisiform bone (cm^2^), flattening ratio of median nerve of at the level of the hamate bone, and TCL thicknesses on the cross-section at the level of the hamate bone (cm) between two groups (all *P* > 0.05). However, there were statistically significant differences in both groups with regard to the above parameters between the baseline and postprocedure values (all *P* < 0.05), suggesting the effectiveness of these two treatment methods. Furthermore, there were statistically significant differences with regard to the postprocedure values of above parameters between the two groups (all *P* < 0.05), suggesting the superiority of ultrasound-guided needle release of the TCL plus corticosteroid injection than the single ultrasound-guided needle release of the TCL.

**Table 2:** Ultrasonic evaluation outcomes at baseline and 3 month after the procedure in two groups

	Group A (*N* = 25)		Group B (*N* = 25)	
	Baseline	Postprocedure	Baseline	Postprocedure
Flattening ratio of median nerve at the level of hamate bone	3.45 ± 0.75	3.18 ± 0.56^a^	3.42 ± 0.81	3.30 ± 0.41^a,b^
Cross-sectional area of the MN at the level of pisiform bone (cm^2^)	0.14 ± 0.02	0.10 ± 0.01^a^	0.14 ± 0.02	0.12 ± 0.01^a,b^
TCL thicknesses on the cross-section at the level of hamate bone (cm)	0.31 ± 0.07	0.15 ± 0.06a	0.32 ± 0.04	0.18 ± 0.05^a,b^

^a^, vs baseline, *P* < 0.05; ^b^, vs Group A; Group A, ultrasound-guided needle release of the transverse carpal ligament plus corticosteroid injection; Group B, single ultrasound-guided needle release of the transverse carpal ligament; LR, Left-right; AP, Anteroposterior; MN, median nerve

### Electrophysiological results

Electrophysiological results at 3 months after the procedure in two groups were shown in Table 3. Prior to procedure, there were no statistical significant differences with regard to the baseline DML and SCV between the two groups (all *P* > 0.05). However, there were statistically significant differences in both groups with regard to the DML and SCV between the baseline and postprocedure values (all *P* < 0.05), suggesting the effectiveness of these two treatment methods. Furthermore, there were significant differences with regard to the postprocedure DML and SCV between the two groups (all *P* < 0.05), suggesting that ultrasound-guided needle release of the TCL plus corticosteroid injection is better than the single ultrasound-guided needle release of the TCL.

**Table 3:** Electrophysiologic outcomes at baseline and 3 month after the procedure in two groups

	Group A (*N* = 18 wrists)		Group B (*N* = 21 wrists)	
	Baseline	Posprocedure	Baseline	Postprocedure
DML(ms)	4.73 ± 0.43	4.1 ± 0.37^a^	4.71 ± 0.52	4.4 ± 0.87^a,b^
SCV(m/s)	36.85 ± 1.46	42.63 ± 2.60^a^	37.16 ± 1.07	39.02 ± 1.07^a,b^

^a^, vs baseline, P < 0.05; ^b^, vs Group A; Group A, ultrasound-guided needle release of the transverse carpal ligament plus corticosteroid injection; Group B, single ultrasound-guided needle release of the transverse carpal ligament; DML, distal motor latency; SCV, sensory conduction velocity

## Discussion

Currently, the use of ultrasound for the guidance of injection has been well established. However, a newer technique using ultrasound-guided percutaneous needle release of the TCL has promising results. A few previous studies have used intraoperative ultrasound for release of the TCL. Nakamichi and Tachibana [14] proposed a method using ultrasonography to protect the critical structures when performing a mini-open carpal tunnel release. Rowe et al. [15] and Lecoq et al. [16] performed cadaveric studies to describe the use of intraoperative ultrasound for release of the TCL while not injury to surrounding structures. Ohuchi et al. [17] even described a combined ultrasound-assisted endoscopic carpal tunnel release technique. Chern et al. [18] presented the technique and results of ultrasound-guided carpal tunnel release with a specially made hook knife in patients with CTS. However, this technique is technically demanding and requires substantial training to be proficient in its use. In this study, we designed the method of ultrasound-guided needle release of the TCL with corticosteroid injections and compared the clinical effectiveness of ultrasound-guided needle release of the TCL with and without corticosteroid injection in early-to-middle-stage CTS. We found that the overall excellent and good rate at 3 months after the procedures was 84% in patients receiving combined procedures, whereas it was only 52% in patients receiving single ultrasound-guided release procedure. Most of the patients receiving combined procedures can achieve satisfactory outcomes after the first therapy; however, most of the patients receiving single ultrasound-guided release procedure can achieve satisfactory outcomes only after multiple sessions. These findings suggested the ultrasound-guided needle release of the TCL with corticosteroid injections had better treatment benefit than single ultrasound-guided needle release of the TCL.

Electrophysiological examination is the traditional investigation of choice in diagnosing CTS [2, 6]. It has a high sensitivity and specificity but is uncomfortable for patients and is time-consuming. Ultrasound is a readily available and cheap diagnostic tool. It has the additional benefit of being able to successfully delineate the subtle changes of the median nerve and TCL in patients with CTS [19]. It can effectively measure median nerve cross-sectional area, thickening of the median nerve, and flattening of the nerve within the tunnel [4]. Now, the diagnostic value of ultrasound has been recognized in studies [20–23] and widely used in clinic. In this study, we measured the electrophysiological parameters such as DML and SCV and ultrasound evaluation parameters such as cross-sectional area of the median nerve at the levels of pisiform, the ratio of left-right diameter to anteroposterior diameter at the levels of the hamate bone, and the thicknesses of TCL on the cross-section at the level of the hamate bone at baseline and 3 months after the procedures. We found that there were statistically significant differences in both groups with regard to the DML, SCV, flattening ratio of median nerve at the level of the hamate bone, cross-sectional area of the median nerve at the level of the pisiform bone, and the thickness of TCL on the cross-section at the level of the hamate bone between the baseline and postprocedure values. These outcomes suggest the effectiveness of both measures. In addition, we found that there were significant differences with regard to the above electrophysiological parameters and ultrasound evaluation parameters after the procedures between the two groups. These findings showed that ultrasound-guided needle release of the TCL with corticosteroid injection is better than the single ultrasound-guided needle release of the TCL. These findings may be explained that needle release of TCL can relieve adhesion and compression, reduce edema of the entrapped nerve, promote blood circulation, and promote the axonal regeneration while the corticosteroids have anti-inflammatory actions and can reduce the inflammatory infiltration and effusion, inhibit the connective tissue proliferation, release the adhesion, inhibit fibroblast proliferation, accelerate the collagen degradation, and reduce the scar formation.

Attention should be paid to the following points during our procedure: (1) The surgeon should have adequate training and expertise in musculoskeletal sonography and ultrasound-guided procedures. Ultrasound-guided needle release of the TCL is technically demanding: the surgeon’s nondominant hand must steadily control the transducer while the dominant hand manipulates the puncture needle under ultrasound guidance. Therefore, we recommend that to prepare for the clinical use of ultrasound-guided needle release procedures, the surgeon can train himself to hold steadily and manipulate skillfully the transducer with their nondominant hand. (2) Before the needle puncture, ulnar artery, ulnar nerve, radial artery, and radial nerve should be verified. During the process of needle puncture, care should be taken to avoid injury to the median nerve and radial and ulnar sides of nerve and blood vessels. (3) Given that the small puncture space of the carpal tunnel, adequate dorsiflexion of the wrist should be performed to increase the puncture space. (4) On attempting to needle puncture, resistance may be felt preventing progress at early stage since the TCL has its inherent high tenacity. The TCL will be suggested to be adequately released once the needle was able to be passed easily through the ligament.

This study has several limitations. First of all, postoperative electrophysiological data were not obtained from all patients. This may be partly because some patients are not willing to perform electrophysiological examination after symptom relief. Furthermore, the follow-up period is somewhat short, which is insufficient to assess the recurrence rate. In this study, no recurrence was observed during this follow-up period. Clinically, the most common cause of CTS is overwork of the wrist, and nerve repair often needs several months, and thus, we will ask patients not to overstrain their wrist within the first several months after treatment. However, during these periods, most patients still tend to overwork their wrist due to living and working habits. Therefore, we select the 3-month follow-up, which can exclude the uncontrollable factor of overwork. Since this minimally invasive technique can be popularized at outpatient clinic, it is worth recommending due to it can relieve pain, improve life quality and facilitate early return to work for the early-to-middle-stage CTS patients. Nevertheless, prospective randomized trials with long-term of follow-up are warranted to confirm the efficacy of our techniques. Moreover, patients included are those who are fail to respond to conservative measures such as splints, non-steroidal anti-inflammatory medication, exercise/therapy, and/or corticosteroid injections and those who did not point to surgical indications. Therefore, future studies are still needed to investigate the optimal populations of this procedure.

## Conclusions

Both techniques are effective in treating CTS. Ultrasound-guided needle release of the TCL with corticosteroid injection had better treatment benefits than single ultrasound-guided needle release of the TCL in treating CTS. Further randomized controlled trials are needed to validate the efficacy of this technique.

## Abbreviations

CTS: Carpal tunnel syndrome
DML: Distal motor latency
NSAIDs: Non-steroidal anti-inflammatory drugs
SCV: Sensory conduction velocity
SD: Standard deviations
TCL: Transverse carpal ligament
